# Integration of palliative care in services for children with life-limiting neurodevelopmental disabilities and their families: a Delphi study

**DOI:** 10.1186/s12913-020-05754-w

**Published:** 2020-10-08

**Authors:** Suzanne Guerin, Gemma Kiernan, Eileen Courtney, Regina McQuillan, Karen Ryan

**Affiliations:** 1 School of Psychology, University College Dublin, Dublin, Ireland; 2 School of Nursing, Psychotherapy and Community Health, Dublin City University, Dublin, Ireland; 3 St Francis Hospice, Dublin, Ireland

**Keywords:** Life-limiting neurodevelopmental disability, Children and families, Services, Delphi study

## Abstract

**Background:**

The aim of this study was to explore expert professionals’ opinions on service provision to children under six with life-limiting neurodevelopmental disabilities (LLNDD), including the goals of care and the integration and coordination of palliative care in general and specialist services.

**Methods:**

A Delphi design was used with three questionnaire rounds, one open-ended and two closed response rounds. Primary data collected over a six-month period from **e**xpert professionals with five years’ (or more) experience in pediatric, intellectual disability and/or palliative care settings. Ratings of agreement and prioritization were provided with agreement expressed as a median (threshold = 80%) and consensus reported as interquartile ranges. Stability was measured using non-parametric tests.

**Results:**

Primary goals of care were achievement of best possible quality of life, effective communication and symptom management. Service integration and coordination were considered inadequate, and respondents agreed that areas of deficiency included palliative care. Improvement strategies included a single care plan, improved communication and key worker appointments.

**Conclusions:**

The findings suggest that services do not serve this group well with deficiencies in care compounded by a lack of information on available services and sub-optimal communication between settings. Further research is needed to develop an expert-based consensus regarding the care of children with LLNDD.

## Background

Appropriate healthcare services are vital for the health and wellbeing of children with life-limiting neurodevelopmental disabilities (LLNDDs). LLNDDs include conditions where there are limitations in the functioning of the brain and/or neuromuscular system, encompassing congenital or acquired conditions, with a variety of neurological, genetic or metabolic aetiologies, that limit the lifespan of the individual [1, 2]. While children in this group may not receive a formal diagnosis, common conditions include cystic fibrosis, muscular dystrophy, severe cerebral palsy, and chromosomal abnormalities [3].

Children with LLNDDS are generally cared for at home, though at times they may need hospital and hospice-based care. Complex medical regimes, dependence on technology and the alternation of medical crises and periods of relative stability necessitate that these children and their families have ongoing contact and interaction with many different healthcare agencies and services [3–5]. The complexity of providing this means that there may be tensions in the goals of care between disparate service providers, particularly in relation to cure-oriented interventions and palliative care [6], whilst service provision may face many unique challenges given this group are at the intersection of disability and palliative care contexts.

There is a growing body of research focusing on the provision of services for children with complex disabilities and life-limiting conditions in general. Studies from healthcare professionals’ perspectives have tended to focus more on experiences of providing services, for example, highlighting the stress experienced and the coping strategies and supports used [7]. However, some studies of healthcare professionals’ views of service provision including the goals of care have been undertaken. Bergstraesser et al. [8] explored the perceptions and needs of pediatric healthcare professionals in Switzerland, including physicians, nurses, and associated healthcare professionals providing care to children with palliative care needs, particularly at the end of life. They recommended specialized pediatric palliative care teams and identified the importance of training for healthcare professionals on issues such as symptom management, quality of life and providing comfort. Also highlighted was the need to provide direct support for families during and beyond the illness of their child, coordination of care, interdisciplinary co-operation and the funding for services.

Given the life-limiting nature of these conditions, palliative and end of life care can be a key part of an integrated service of this group. For example, Graham and Robinson [6] put forward a model for end of life care for children with LLNDDs and proposed that the primary goal of end-of-life care for these children must be to integrate palliative and curative care approaches with the emphasis on quality of life. Further research is required to ascertain healthcare professionals’ perspectives about what constitutes optimal service delivery for children with LLNDDs throughout their lives. The present study conducts a Delphi exercise to further examine professionals’ views on this this issue.

It’s important to recognise the context in which the present study is conducted. Children with neurodevelopmental disability represent the largest group of children with chronic disease in Ireland. The country has a national complex disability prevalence of 4%, with approximately 45,325 children aged 0–19 years in need of specialized care [1]. Disability services in Ireland, which provide supports to children with LLNDD and their families, are recognized to be a chronically under-resourced area of health. Services have evolved in an uncoordinated, ad hoc manner resulting in a lack of integration leaving some children and their families with little or no access to care. Palliative care services are less developed than disability services, and have traditionally been informed by the Palliative Care for Children with Life-limiting Conditions in Ireland national policy [9]. However broader disability services are currently being reconfigured through the Progressing Disability Services for Children and Young People (PDSCYP) program [10] and the recommendations of the National Model of Care for Paediatric Services in Ireland [11]. Reconfiguration aims to provide maximum levels of care as close to home as possible, and to ensure that children with complex needs can easily access specialist support. Services will be organized according to a hub-and-spoke model. In this way, delivery of the majority of care is facilitated locally though integrated service networks with outreach support from tertiary and quaternary pediatric subspecialties. While these recommendations postdate the present study, the process of implementation has been varied, and consistent service delivery has not yet been achieved.

Within this changing context, here is a need for explicit goals of care for this group that bring together disability and palliative care services, as well as a need to explore how services can be improved. Therefore, the aim of the study is to gather expert opinion from health professionals regarding care and services for children with LLNDDs in Ireland. While there are many relevant stakeholders in this area including parents and practitioners, this study builds on previous work with these groups as discussed above [3, 5, 7, 8] by adding the insights from experts. This study addresses the following research questions -

- How do experts rate the integration and coordination of services (including palliative care) for these children and their families?
- What are the goals of care for children with LLNDD and their families?
- What changes are necessary to improve services for this group?

## Methods

Recognizing the multiple specialisms that contribute to care for children with LLNDDs and their families, this Delphi study draws on experts from general pediatric services, intellectual disabilities and palliative care. During repeat rounds, panelists were invited to confirm/modify previous responses in the context of the group opinion, until consensus was reached [12]. The value of Delphi was its ability to generate ideas and work towards consensus from expert panelists.

Initially, three qualitative interviews were conducted to assist the formulation of the questionnaire (see Supplemental file 1 for interview guide). Initial views explored the goals of care for the target population, aspects of current services and what changes were required to improve services. Participants in initial interviews were not included in subsequent rounds. Interviews were followed by three questionnaire rounds, one open-ended, two closed, to reach consensus on the target issues.

### Selection of expert panel

Sampling involved purposeful sampling [13] from general pediatric, intellectual disability and palliative care services. This ensured a representative range of views. Key characteristics of the panel were expertise and credibility. Experts held a professionally recognized health-related qualification with no less than 5 years’ experience providing care to children with LLNDD and their families. Credibility was demonstrated by the fact that individuals were identified as experts by peers. Individual identified were then asked to nominate other experts who also met the criteria.

The target for the expert panel was 20, with 24 individuals initially identified. Many names were identified by several sources, reinforcing their inclusion as experts. All 24 individuals were approached to participate, with each participant assigned a unique identification code. This ensured that inter-round feedback could be tracked, and the researcher could ensure there was sufficient mix of responses to each round. Nineteen experts agreed to participate. One panelist subsequently withdrew before the study commenced, and a further five returned no questionnaires. Thus, the final panel consisted of 13 expert members. Based on Endacott et al. [14] and Wierner et al. [15] all members of the expert panel were invited to participate in each round, regardless of their response in the previous round.

The experts represented several disciplines and services providing care to children with LLNDD and their families. Six members worked in statutory services, five in voluntary services and the remaining two in independent charity services. Six members came from the general pediatric services, five from ID services and the remaining two from palliative care services. Five panelists were from nursing/therapy disciplines, three from medicine, four were service managers/coordinators and one from social work. This ensured a comprehensive representation of experiences, views and opinions. Although comparatively small, the experience of the participants ensured a sufficient panel for a credible Delphi [16, 17].

### Questionnaire development

The Round 1 questionnaire (Supplemental file 2) used open-ended questions generated by the research team from the existing literature to allow key issues to be identified. This included views on the coordination and integration of current services (including palliative care); the goals of care, and the changes to services necessary to improve care to children and their families. The Round 1 questionnaire was reviewed by the three experts for face/content validity before being distributed. The interviews and textual responses to Round 1 (R1) were analyzed using content analysis [18], informing the development of the closed questionnaire for Round 2 (R2) and 3 (R3). The resulting questionnaire used in R2 and R3 (Supplemental file 3) included three sections.

- The coordination and integration of current services; 12 items - five-point Likert scale (1–Strongly Disagree to 5–Strongly Agree).
- The goals of care for this group; 13 items - ranked 1 = most important goal of care.
- Service-related changes required to improve services; 18 items - ranked 1 = most important change.

The questionnaire was piloted with the expert interviewees to ensure face validity and no changes were made. The questionnaire included all items in R2 and R3, including those on which agreement had been reached in R2. This ensured that comprehensive feedback was provided to the panel and allowed for the assessment of response stability between R2 and R3.

### Data analysis

Quantitative data from R2 and R3 were analyzed using SPSS®. Agreement included the extent to which respondents agreed with the issue and the extent to which respondents agree with each other [17, 19]. Level of agreement for individual questionnaire items used the median, as it is less sensitive to extreme scores [20] and appropriate when data are ordinal [21]. Although a systematic review by Diamond et al. [22] reports 75% as the median threshold to define consensus in multidisciplinary studies, given the small sample in this study the threshold for agreement in this study was set at 80% [23] and disagreement was also explored. The level of consensus (group agreement) was expressed as the interquartile range (IQR), defined as IQR ≤ 1 for service descriptors [24], and ≤ 2 for the rank ordering of goals/priorities [25]. Stability was defined in accordance with Howitt and Cramer [26], with the Wilcoxon Signed Rank Test used to establish group response stability with significance at 0.05 and items with *p* ≥ 0.05 were considered stable. Respondents were provided with summary statistics between rounds, including mean scores for rank order items, and their individual scores from the previous round were also included. In R3, participants identified as outliers (those in the lower and upper quartiles from the previous round) were asked to provide written justification for their views, allowing areas of disagreement to be explored.

## Results

Response rates for individual Delphi rounds were variable, ranging from 69% in R1, to 100% in R2 and 92% in R3, with 46% completing all three rounds. Although the response rate for R1 did not achieve Sumison’s [27] 70% standard, non-responders were spread amongst the subgroups, thus minimizing potential bias. Given that R1 was used to inform the development of the closed questionnaire used in R2 and R3, the reporting of the findings in this paper focuses on the quantitative analysis of these questionnaire rounds, in particular on changes between the rounds.

### Integration and coordination of services

Responses to statements regarding integration and coordination of current services (including palliative care) are presented in Table 1, which includes findings from both rounds, allowing for changes and response stability to be compared. Wilcoxon’s Signed Rank Tests are also reported, with *p* ≤ 0.05 set as the significance level. As this section of the questionnaire asked experts to rate their agreement with the statements the overall level of agreement at R3 is also reported (reflecting the combined frequency of agree/strongly agree responses).

**Table 1:** Responses to statements regarding the integration and coordination of current services including palliative care

Service Integration and Coordination	R2 Mean	R2 Median	R2 IQR	n	R3 Mean	R3 Median	R3 IQR	n	Agreement	W	*p*
1. There is poor communication between acute services and community-based services in the care of these children**	4.00	4.50	1.75	12	4.08	4.00	1.00	12	83%	−1.41	0.16
2. General Practitioners lack the experience and expertise necessary to deal effectively with these children	3.92	4.00	2.00	12	4.08	4.00	1.75	12	75%	−1.00	0.32
3. There is poor coordination and integration of services involved in the care of these children and their families	4.08	4.50	1.75	12	4.00	4.00	1.75	12	75%	−0.82	0.41
4. Acute services are not aware of the range of services that are available to children and their families in the community**	3.54	4.00	1.00	13	4.00	4.00	0.00	12	92%	−1.34	0.18
5. Families receive conflicting information about their child from different services	3.62	4.00	1.00	13	3.58	4.00	1.00	12	68%	−1.00	0.32
6. There is collaboration between the different services regarding the goals of care for these children	2.54	2.00	3.00	13	2.00	2.00	2.00	11	18%	−1.34	0.18
7. Medical teams lack interest in these children because of their limited prognosis	2.75	2.00	2.75	12	4.42	2.00	1.75	12	25%	0.00	1.00
8. Children with life-limiting neurodevelopmental disabilities often undergo futile investigations and procedures	3.50	3.50	2.50	12	3.42	3.50	1.00	12	50%	0.00	1.00
9. Medical staff are reluctant to discuss the fact that children are “life-limited” with parents	3.92	4.00	1.75	12	3.67	4.00	2.75	12	67%	0.00	1.00
10. Medical teams fail to recognize the palliative care needs of the child	3.85	4.00	2.50	13	3.83	4.00	2.00	12	67%	−1.00	0.32
11. Palliative care is only considered late in the child’s condition or in crisis management**	4.50	4.50	1.00	12	4.25	4.00	1.00	12	92%	0.00	1.00
12. Access to specialist palliative care services is readily available if it is required	2.46	2.00	3.00	13	2.42	2.00	1.50	12	25%	−1.41	0.16

Scale: 1 Strongly Disagree – 5 Strongly Agree; ** Indicates Agreement and Consensus achieved in R3 Response Stability achieved on all items in R3

Examining statements relating to the integration and coordination of services (items 1–6 Table 1), expert agreement and consensus was reached on only two statements, and only group consensus on a third. Wilcoxon’s Signed Rank Test indicated stability of responses between R2 and R3 for all items. The group agreed that there are some difficulties relative to the interface between acute and community-based services, specifically that there is poor communication between the two, and that acute services are not aware of the range of services available to children and their families in the community. Two other statements approached, but did not achieve, the threshold of agreement. Seventy-five percent of the panel (*n* = 9) agreed that there is poor coordination and integration of services, and the same proportion (75%) agreed that General Practitioners lack the experience and expertise required to care for children with LLNDD.

Within the context of integrated care, propositional statements related to the provision of palliative care to young children with LLNDD and their families achieved the lowest level of agreement in this study. Of the six statements, only one achieved both agreement and consensus in the final round (Table 1). Wilcoxon Signed Ranks Test indicated stability of responses with no statistically significant change to scores between R2 and R3. Specifically, the expert panel agreed that in palliative care is only considered late in the child’s condition or in cases of crisis management. The IQR for four other items was reduced between R2 and R3, indicating a move towards consensus, but the IQR for the statement “medical staff are reluctant to discuss the fact that children are life-limited with parents” increased from 1.75 in R2, to 2.75 in R3. One other statement approached the study’s agreement threshold, with 75% of the group (*n* = 9) disagreeing that access to palliative care is readily available if it is required.

Group opinion on the approach adopted by health professionals was divided, although generally the trend was towards a negative perception, which was indicated by comparing the percentage of the group who agreed with the percentage who disagreed excluding the percentage of “not sure”. Fifty percent of the expert panel (*n* = 6) agreed or strongly agreed that children often undergo futile interventions compared with 17% who disagreed or strongly disagreed (*n* = 2). Sixty seven percent of the group (*n* = 8) agreed that medical teams fail to recognize the palliative care needs of the child, with the same proportion agreeing that there is reluctance on the part of medical teams to discuss with parents the fact that the child’s condition is life-limiting. Finally, 67 % of the group (*n* = 8) disagree that there is a lack of interest in these children because of their limited prognosis.

### Goals of care

The goals of care are presented in Table 2 which includes the findings from both rounds allowing for changes and response stability to be compared. Wilcoxon’s Signed Rank Tests are also reported, with *p* ≤ 0.05 set as the significance level.

**Table 2:** Respondents’ rank order of goals of care for children with LLNDDs in Rounds 2 & 3

Goal	R2 Priority	R2 Mean	R2 Median	R2 IQR	n	R3 Priority	R3 Mean	R3 Median	R3 IQR	n	W	*p*
Achievement of the best possible quality of life for the child**	1	1.92	1.00	1.00	13	1	1.17	1.00	0.00	12	−1.34	0.18
Open & honest communication with the family**	3	4.38	4.00	2.50	13	2	3.75	3.00	1.75	12	−1.60	0.11
Achievement of the child’s full potential within the limits of the illness	4	5.15	5.00	6.00	13	3	3.90	3.50	3.00	12	−1.60	0.11
Optimum symptom management**	5	5.69	5.00	5.50	13	4	4.00	4.00	2.00	12	−1.86	0.06
The child is cared for at home	2	4.15	4.00	4.0	13	5	4.08	3.00	3.5	12	0.00	1.00
Parents are supported with the provision of care	7	6.54	6.00	2.50	13	6	6.75	7.00	2.75	12	−1.34	0.18
Promotion of normality for the child and family	6	6.08	5.00	6.00	13	7	6.83	6.00	3.75	12	−1.07	0.29
Provision of appropriate respite	8	6.62	7.00	5.00	13	8	7.25	7.50	3.50	12	−0.45	0.66
The family continues to function as a unit and enjoy life	9	6.85	5.00	5.00	13	9	7.75	8.50	3.75	12	−2.03	0.04
Inappropriate medical interventions are minimized	11	8.00	8.00	4.50	13	10	8.58	9.50	3.00	12	−1.22	0.22
Achievement of a seamless web of care	10	7.54	7.00	2.50	13	11	8.67	9.00	3.75	12	−1.60	0.11
The child’s life is prolonged**	12	11.54	12.00	1.00	13	12	11.50	12.00	0.75	12	0.00	1.00
The family is provided with the hope that things will get better**	13	11.69	13.00	1.00	13	13	11.83	13.00	1.00	12	−1.34	0.18

**Indicates Consensus achieved in R3

At R3, there was relatively little change to the rank order assigned to goals of care from R2. Five goals retained their priority status, while an additional seven moved up or down one ranking in R3. The priority of the goal “The child is cared for at home” demonstrated the greatest shift between rounds falling from second place in R2 to fifth place in R3, although the Wilcoxon Signed Rank Test demonstrated that there was no significant difference in the mean scores for this goal between rounds (W = .00, *p* = 1.00).

While there was some movement in terms of priority awarded, the five highest ranking goals from R2 retained their top five ranking in R3. Three of these five goals achieved consensus in R3; “achievement of the best possible quality of life for the child” retained its number one priority; “open and honest communication with the family” increased from third priority in R2 second priority in R3; and “optimum symptom management” increased from fifth priority to fourth priority in R3.

Two of the top five goals of care identified by the expert panel failed to reach consensus in the final round. Although ranked as the third highest priority, “achievement of the child’s full potential within the limits of the illness” did not achieve consensus (IQR = 3). Three panelists provided commentary. For two the goal was synonymous with access to appropriate education and was inextricably linked with the issue of overall quality-of-life for the child. Similarly, although identified as the fifth ranked goal, “the child is cared for at home” did not achieve consensus in this round (IQR = 3.5). Exploration of the commentaries provided suggests that while in many cases this is an important goal, in others it is neither desirable nor achievable.

The lowest ranking goals “the child’s life is prolonged” and “the family is provided with the hope that things will get better” retained their position between R2 and R3, and both achieved consensus in R3, suggesting that the expert panel agreed that these were not priority in the care of young children with LLNDDs and their families.

Overall, the IQR for eleven of the goals of care were reduced between R2 and R3, demonstrating a move towards consensus. This did not hold true however for two goals; “parents are supported with the provision of care” and “achievement of a seamless web of care”. These demonstrated a small increase in IRQ of .25 and 1.25 respectively. Wilcoxon’s Signed Rank Test indicated stability of responses between R2 and R3 for 12 of the goals. The only goal not to achieve response stability was “the family continues to function as a unit and enjoy life” (W = − 2.03, *p* = .04).

### Priorities for improving services

Six change priorities for improving services retained their original ranking between R2 and R3 (Table 3), with an additional ten moving up or down one ranking between the rounds. Wilcoxon Signed Rank Test demonstrated that there was no significant difference in the mean scores for many of the priorities for improving services between rounds; with significant values reported for a single care plan for use across all services (W = − 2.23, *p* = 0.03) and a national directory of services (W = − 2.04, *p* = 0.04).

**Table 3:** Respondents Rank Order of Priorities for Improving Services to Children with LLNDs and their Families Rounds 2 & 3

Required Service Change	R2 Priority	R2 Mean	R2 Median	R2 IQR	n	R3 Priority	R3 Mean	R3 Median	R3 IQR	n	W	*P*
A greater level of communication between all the health professionals involved in the care of the child**	3	5.77	4.00	7.00	13	2	3.75	3.00	1.75	12	− 1.47	0.14
A key worker available to every family**	2	4.92	3.00	8.50	13	3	3.92	2.50	1.00	12	−0.96	0.34
A single care plan for use across all services**	1	4.38	3.00	5.00	13	1	2.75	1.00	1.00	12	−2.23	0.03
A greater level of coordination & integration of the services involved in the care of the child	5	7.08	6.00	7.00	12	4	5.50	5.00	3.50	12	−0.94	0.34
A single point of contact for information for families	7	8.62	8.00	8.00	13	10	9.33	9.00	6.50	12	−0.38	0.71
Less bureaucracy with regards to the family’s entitlements	13	11.00	12.00	7.50	13	14	11.83	12.50	6.25	12	−0.55	0.58
Access to palliative care in a timely and efficient manner	6	7.85	5.00	3.00	13	6	7.33	6.00	5.25	12	−0.55	0.58
Parent held medical records	18	12.92	15.00	9.50	13	17	14.83	17.50	7.75	12	−1.83	0.07
A national directory of services	17	12.85	13.00	2.00	13	18	14.92	15.00	4.75	12	−2.04	0.04
Improved education for community-based health professionals	14	11.46	13.00	5.50	13	13	11.75	13.00	5.50	12	−0.92	0.36
A specialist pediatric palliative care consultant to act as a resource when required	4	6.46	5.00	4.00	13	5	7.08	5.50	6.25	12	−0.68	0.50
A formal care coordinator in every HSE area	11	9.54	10.00	9.00	13	11	10.75	11	6.5	12	−0.68	0.50
Medical priority status in A& E and OPD departments	12	9.77	9.00	8.50	13	12	10.92	12.00	8.25	12	−1.60	0.11
The development of community based pediatric palliative care teams	8	8.85	8.00	9.50	13	9	9.33	7.50	7.25	12	−0.37	0.72
Improved respite facilities	9	8.69	7.00	7.50	13	8	9.17	9.50	4.00	12	−0.18	0.85
A less protracted system for ordering essential equipment	16	12.38	12.00	9.00	13	16	12.17	12.50	6.75	12	−.32	0.76
Improved communication between acute and community services	10	9.23	9.00	10.00	13	7	8.58	9.00	6.00	12	−0.68	0.47
National standards of care	15	11.54	13.00	8.50	13	15	12.00	15.50	9.5	12	−2.03	0.04

**Indicates consensus achieved in R3

While there was some movement in terms of priority awarded, the five highest ranking priorities for change from R2 were retained as the five highest ranking priorities in R3. Three of these achieved consensus in the final round. These included “a single care plan for use across all services” ranked as the highest priority change, “a greater level of communication between all the health professionals involved in the care of the child” ranked second highest priority, and “a key worker available to every family” ranked third.

Although ranked fourth and fifth respectively, neither “a greater level of coordination and integration of services” nor “a specialist pediatric palliative care consultant to act as a resource when necessary” achieved consensus in R3 with IQRs of 3.5 and 6.25 respectively, indicating a wider range of disagreement amongst the group with regards to the final ranking of these priorities. The IRQ was reduced for the remaining 13 items in this subscale in R3, suggesting a move towards consensus in this round. However, overall the range of IQR remained wide (3.5–9.5) which suggests that, excluding those items which were ranked as being the three most important priority changes to services, and on which consensus was reached, there was relatively little agreement amongst the panel as to the priority service changes should take to improve the care provided to children with LLNDD and their family.

## Discussion

A key aim of the study was to examine expert opinion on the integration and coordination of current services including palliative care for children with LLNDD and their families in Ireland. The findings suggest that services do not serve this group well, with deficiencies in care compounded by a lack of information on available services and sub-optimal communication between settings. These findings reflect previous research with professionals in other pediatric settings [8, 28]. The achievement of quality of life (rather than prolongation of life) was strongly articulated as the primary goal of care for children with LLNDD in keeping with an overall palliative approach to the care of these children. However, it was the opinion of the panel that palliative care is considered relatively late in the child’s condition or in crisis management. This is at odds with the current trend towards early integration of palliative for adults and children seen internationally. Despite the clarity of these statements, the lack of consensus when considering other aspects of palliative care was notable, pointing to different experiences of and opinions regarding provision in Ireland. Diversity of opinion was evident when considering such matters regarding communication with parents, recognition of palliative care needs and adequacy of access to specialist palliative care.

Perhaps unsurprisingly, the panel’s recommendations regarding priority changes that would improve care are closely tied to statements that reached consensus in the early sections of the Delphi questionnaire. The top three priority changes to improve current services (i.e., a single care plan, better communication, assignment of key workers) are consistent with the expert panel’s agreement that there is inadequate information, poor communication and suboptimal access to services. Previous research has suggested that key-worker availability would improve coordination and integration of services to the child and family [28–30] and it appears that the expert panel were of a similar opinion.

Even though quality of life was felt to be the primary goal of care for children with LLNDD and that inadequacies in palliative care provision were noted, strategies to improve palliative care provision did not gain consensus. The ranking that the strategies were given in the service improvement priority list indicated that some respondents did regard them as having some importance. Nevertheless, this was not the opinion of a sufficient majority. Our research did not explore reasons why those recommendations did not reach consensus, but barriers to the integration of palliative care in children’s care are well described in the literature [31–33]. It is possible that members of the expert panel share some of those viewpoints regarding the place of palliative care in children’s care, or it may be that they did not consider the strategies presented in the Delphi were the right ones to advance service provision. Delphi studies can help uncover social dimensions to decision-making and the lack of agreement on palliative care highlights an area that merits further exploration.

The centrality of a coordinated and multiagency approach to the planning and delivery of care and support to all children with palliative care needs has been documented [34, 35, 1]. Campbell [36] identifies that in the context of disability services the highest quality ratings are achieved when there is evidence of the use of multidisciplinary integrated care pathways which clarify expected steps and outcomes. Similarly, the priority awarded to a keyworker for every family is consistent with the panel’s agreement that the current lack of this resource results in the ad hoc delivery of services. This is consistent with findings of previous research which suggest that key-worker availability would improve coordination and integration of services to the child and family [28–30].

A key issue here appears to be the inconsistency between the panel’s opinion on service coordination and the proposal of “improved service coordination and integration” as a change that would improve services and the opinion of health professionals in Quinn et al’s [28] study of professionals’ opinions about service integration and coordination for a generic population of life-limited children in the context of specialist palliative care. Another inconsistency relates to the panel’s proposal for the appointment of a specialist palliative care consultant as a priority change to services that would improve care. This appears inconsistent with the panel’s opinion on the current provision of palliative care to children with LLNDD, especially the panel’s failure to agree on issues of access to specialist palliative care. One explanation for this may be the difference between having access to an adult specialist palliative care service adapted to the needs of a child with a LLNND as is currently available, and access to a specialist pediatric palliative care service, which would be focused exclusively on the needs of the child and family, as is the proposed change to current services. In addition to specialist services, there was no consensus (despite high levels of agreement) on the potential contribution of GPs to care for this group. In the context of a lack of agreement of both generalist and specialist support for these services, there may be a vacuum about how to structure services.

While it has been clearly established that children’s needs from palliative care differs from that of adults [37], there are benefits to examining how the issues raised above have been considered in the adult palliative care literature. Looking to this literature, it seems that the issues of late identification and a lack of integrated services are also present here [38]. However, several initiatives are evident internationally that have sought to addresses these issues in adult settings. Gomez-Batiste et al. [38] report a number of European initiatives that have worked to address these issues in adult settings, including the issue of tools to promote early identification and prevalence of need and proposals for improving the delivery of palliative care approach in health service settings. Despite these initiatives, it is clear that there remain significant outstanding issues in adult care. Nevertheless, developments along these lines in children’s services would no doubt begin to address the issues identified in the present study. Indeed, the need to integrate children’s palliative into pediatric care is noted by the World Health Organisation [37].

### Strengths and limitations

The findings of this study should be considered in the context of its strengths and limitations. There is little definitive guidance to be found in the literature with regards to the decisions made during a Delphi study, and although the decisions made, and the rationale for them, have been made clear in this study, it reflects the positive and negative contributions of the Delphi method.

The study used a relatively small expert panel when compared to the panel size in much published literature, however this may reflect the small expert pool available in Ireland. In this study, every effort was made to ensure this was a comprehensive panel, which included all services involved in the care of children with LLNDD and their families, nonetheless the findings should be considered in the context of the panel size. Nevertheless, the discrepancies between the perception of service coordination and proposed changes to services may be associated with the small number of experts. It is also possible that poor coordination of services in not a national problem and that service integration and coordination is worse in some areas than in others.

It is also difficult to directly compare the response rate with other published literature due to ambiguity in what is being reported. Gibson, Koepsell, Diehr and Hale [39] reported a 64% response rate, while Butterworth and Bishop [40] report a response rate of 61%, however it is not clear whether this figure represents the respondents who completed all rounds. Sumsion [27] suggests a minimum of 70% for each round. However, commonly this minimum response rate is not achieved, with a range of 40–65% reported in the literature [14, 41, 42, 15]. This study met Sumsion’s standard for all but the first round.

### Recommendations

It is the explicit and stated aim of Irish health policy to provide health services to all who need them. Despite these stated visions and aspirations, developing systems of services to best meet the needs of young children with LLNDD and their families continues to represent a significant challenge for all services and agencies involved in the care of this population. As noted in the Introduction, data collection for this study preceded several developments in policy with implications for supports to children with LLNDDs and their families. The PDSCYP program [10] and the recommendations of the National Model of Care for Paediatric Services in Ireland [11] aims to maximize access to specialist care close to home for children with complex needs. However, the need to fully implement these policies has been noted by researchers in the area [43]. So, while these documents provide a framework within which many of the issues noted in the present study can be addressed, the process of operationalizing change is ongoing. While not explored in this study, further research exploring healthcare professionals views regarding service improvements would further contribute to this process.

The present study’s findings highlight several possible recommendations for the care and support of children with LLNDD and their families, with the goal of achieving the best possible quality of life for the child and achievement of their full potential within the limits of the illness. With the focus on coordinated and integrated services noted in the findings, the practical operation of assigning a key worker to every family and developing a single care plan for the family, would contribute to achieving this. This would be further complemented by a focus on open and honest communication with the family and between health care professionals. Indeed, both elements are part of the PDSCYP program and reflect the emphasis of this program on family centered care [44]. Reflecting on the other priorities of care reported in this study, the findings highlight the potential contribution of palliative care for this target group. The need for optimum symptom management, allowing the child to be cared for at home, would be supported by increased accessed to specialist pediatric palliative care as a resource when required. There is no doubt that pediatric palliative care services in Ireland are not as developed as those available for adults, but developments in service provision such as those described in this article may ensure additional support. However, even in countries where pediatric palliative care is more developed [45], there is no guarantee that these services would be part of an integrated service network accessible to children with LLDNN and their families.

## Conclusion

The expert opinion of this Delphi panel is that currently, services to young children with LLDNN and their families are under-funded and under-resourced, with definite gaps in some areas of service provision as well as poor communication between acute and community-based services. While the expert panel agrees on what the goals of care for this population of children and their family are, there is less consensus regarding the changes to current services that are required to achieve these goals and improve services to this population of children and their families. This makes acting to improve services to young children with LLNDD even more complex, though the findings of this study do offer specific recommendations that would contribute to more effective services.

## Supplementary information

**Additional file 1: Supplemental file 1.** Delphi Interview

**Additional file 2: Supplementary file 2.** Round 1 Questionnaire

**Additional file 3: Supplementary file 3.** Round 2 and 3 Questionnaire

## Data Availability

Data collection materials are available as supplementary files for this paper. The raw data from this study are no longer available, due to the limits required by ethical approval. However additional information is available on the analyses conducted. Please contact Suzanne.Guerin@ucd.ie for access to this additional information.

## Abbreviations

LLNDD: Lifel-imiting Neurodevelopmental Disability
HSE: Health Service Executive
PDSCYP: Progressing Disability Services for Children and Young People
R1: Round 1
R2: Round 2
R3: Round 3
IQR: Interquartile Range
