# Supplementary material for: Integration of palliative care in services for children with life-limiting neurodevelopmental disabilities and their families: a Delphi study
Source: BMC Health Serv Res. 2020 Oct 8;20:927. doi: 10.1186/s12913-020-05754-w (PMC7545942; doi:10.1186/s12913-020-05754-w)
Supplement: Supplementary file 1 — Additional file 1: Supplemental file 1. Delphi Interview [file 12913_2020_5754_MOESM1_ESM.docx]

Supplemental File 1 - Delphi Interview

*Interview Topic Guide*

This interview is concerned with the following topics

(1) Can you begin by explaining what, in your expert opinion, are the goals of care / the principles that should govern care when a child has a life-limiting neurodevelopmental disability?

· Explain why each goal is important / relevant in the care of this population.

(2) In your opinion how do current services meet those goals?

· Which goals are achieved and which are not?

· If goals are not achieved in the context of current services why do you think this is so?

(3) What changes are necessary to current services to improve the care of children with life-limiting developmental disabilities and their families?

· What is required in order to achieve these changes?
