# Supplementary material for: Integration of palliative care in services for children with life-limiting neurodevelopmental disabilities and their families: a Delphi study
Source: BMC Health Serv Res. 2020 Oct 8;20:927. doi: 10.1186/s12913-020-05754-w (PMC7545942; doi:10.1186/s12913-020-05754-w)
Supplement: Supplementary file 2 — Additional file 2: Supplementary file 2. Round 1 Questionnaire [file 12913_2020_5754_MOESM2_ESM.docx]

**Supplementary File 2 – Round 1 Questionnaire**

**Delphi Round 1**

Instructions for Responding to Round 1.

***Please read the following information carefully before completing the questionnaire.***

| This study is concerned with the palliative care needs of young children (under the age of six) with life-limiting neurodevelopmental disabilities and their families.  In the context of this study a *“Life Limiting Neurodevelopmental Disability”* is defined as a condition of neurodevelopmental origin for which there is currently no cure and which is likely to lead to the child dying prematurely.  The study defines a “*Palliative Care Need”* as a physical, psychological, social or spiritual need that is present in the context of life-limiting or terminal illness. It is the context of the life-limiting condition that designates a palliative care need, although this does not necessarily imply a requirement for specialist palliative care services.  Please complete this questionnaire in the context of these definitions. |
| --- |

This questionnaire consists of three broad subject areas related to the goals of care for this population of children and their families and the way in which current services meet these goals. Please complete all parts of the questionnaire based upon your own personal opinion and experience (the answers you provide are not taken to represent the views of the services in which you work), and include additional sheets if required.

Each question consists of two parts. The left-hand column asks you to identify a particular issue while the right-hand column asks for additional

information or further explanation. The answers in both columns are important as they provide and additional way of viewing your responses to insure they are not misinterpreted. Thank You.

| **Q.1 Please use the space below to identify what you consider to be the goals of care for children with life-limiting neurodevelopmental disabilities and their families.** | **For each goal you identify please explain how / why you believe it is important to this population.** |
| --- | --- |
|  |  |

| **Q.2 Based upon your previous answer please describe which goals you feel are achieved in the context of current services and which are not.** | **If goals are not achieved in the context of current services what do you think is preventing their achievement?** |
| --- | --- |
|  |  |

| **Q.3 What changes do you consider are necessary to current services to improve the care provided to children with life-limiting developmental disabilities and their families?** | **Please explain what resources would be required in order to achieve these changes.** |
| --- | --- |
|  |  |
