# Supplementary material for: Integration of palliative care in services for children with life-limiting neurodevelopmental disabilities and their families: a Delphi study
Source: BMC Health Serv Res. 2020 Oct 8;20:927. doi: 10.1186/s12913-020-05754-w (PMC7545942; doi:10.1186/s12913-020-05754-w)
Supplement: Supplementary file 3 — Additional file 3: Supplementary file 3. Round 2 and 3 Questionnaire [file 12913_2020_5754_MOESM3_ESM.docx]

**Supplementary File 3 – Round 2 and 3 Questionnaire**

**Delphi Survey – Round 2**

*Exploring the Palliative Care Needs and Delivery of Services to Young Children with Life-Limiting Neurodevelopmental Disabilities and their Families*

Please read the following information before completing the questionnaire.

| Thank you for completing the first round of this Delphi.  This second round questionnaire is based on the analysis of information provided in the previous round together with information obtained from a national survey of parents and interviews conducted with health professionals working in this area.  The format of this round is different to the previous one. This questionnaire is divided into three sections. In sections 1 and 3 you are asked to rank order issues relative to their importance in the overall care of children with life limiting disabilities’ and their families. In section 2 you are presented with a list of statements on which you are asked to rate your agreement using a simple 5 point Likert scale. Instructions for completing each section are provided at the outset. Please read these carefully before completing the questionnaire.  The questionnaire should take no more than 30 minutes of your time to complete. It is vital to the success and credibility of this study to have a high a response rate, so I would ask that you return this questionnaire even if you did not respond to the previous round.  **Please return completed questionnaires in the prepaid enveloped by September 10th.**  Thank you. |
| --- |

Section 1

*This section relates to the goals of care for children with life-limiting neurodevelopmental disabilities and their families.*

Based upon the data collected from parents and the responses from round one the following issues were identified as the important goals of care for children with life-limiting neurodevelopmental disabilities and their families. You are asked to do two things with this list –

1. Please read and review all of the 13 goals on the list. Please rank these goals in order of their importance for the care of these children and families. Assign a weight of one (1) to the most important, two (2) to the second most important etc. until you have completed all 13.
2. Comment, in one or two statements, on any goal(s) that you wish. You may argue in favour of a goal, against a goal, or request clarification. Brevity and clarity will facilitate analysis.

| **Goal Weight** | **Improvement** | **Comment** |
| --- | --- | --- |
|  | - The child is cared for at home. - Achievement of the best possible quality of life for the child. - Provision of appropriate respite services. - Achievement of the child’s full potential within the limits of the illness. - Inappropriate medical interventions are - minimised. - The family continues to function as a unit and enjoy life. - Promotion of normality as much as possible for the child and family. - Open & honest communication with the family. - The child’s life is prolonged. - Optimum management of symptoms. - Parents are supported with the provision of care. - The family is provided with the hope that things will get better. - Achievement of a seamless web of care. |  |

If there are other comments that you would like to add please include them in the space provided on the next page.

| Please use this space if you would like to provide any additional comments on the Goals  of Care listed in Section 2 |
| --- |

Section 2

*This section relates to the current provision of service to children with life-limiting neurodevelopmental disabilities and their families.*

Please read each statement carefully and indicate your agreement by marking the box that best represents your view from 5 = strongly agree to 1 = strongly disagree.

|  | Strongly Agree | Agree | Not Sure | Disagree | Strongly Disagree |
| --- | --- | --- | --- | --- | --- |
| 1. Children with life-limiting neurodevelopmental disabilities and their families are well served by current services. | 5 | 4 | 3 | 2 | 1 |
| 2. A lack of key workers for families results in the ad hoc delivery of services to this population. | 5 | 4 | 3 | 2 | 1 |
| 3. The child and the family are seen as a single unit of care. | 5 | 4 | 3 | 2 | 1 |
| 4. There is a heavy reliance upon charity services to meet the needs of this population. | 5 | 4 | 3 | 2 | 1 |
| 5. There is sufficient psychological support available to parents of children with life-limiting neurodevelopmental disabilities. | 5 | 4 | 3 | 2 | 1 |
| 6. These children often undergo futile investigations and procedures. | 5 | 4 | 3 | 2 | 1 |
| 7. Services are flexible enough to respond to a family crisis at short notice. | 5 | 4 | 3 | 2 | 1 |
| 8. There is poor communication between acute services and community based services in the care of these children. | 5 | 4 | 3 | 2 | 1 |
| 9. Medical teams lack interest in these children because of their limited prognosis. | 5 | 4 | 3 | 2 | 1 |
| 10. Current respite services are sufficient to meet the needs of this population. | 5 | 4 | 3 | 2 | 1 |
| 11. General Practitioners lack the experience and expertise to deal effectively with these children. | 5 | 4 | 3 | 2 | 1 |
| 12. There is sufficient psychological support available to siblings of children with life-limiting neurodevelopmental disabilities. | 5 | 4 | 3 | 2 | 1 |
| 13. There is poor coordination and integration of services involved in the care of these children and their families. | 5 | 4 | 3 | 2 | 1 |
| 14. Medical staff are reluctant to discuss the fact that children are “life-limited” with parents. | 5 | 4 | 3 | 2 | 1 |
| 15. There is good home support for end-of-life care for these children. | 5 | 4 | 3 | 2 | 1 |

|  | Strongly Agree | Agree | Not Sure | Disagree | Strongly Disagree |
| --- | --- | --- | --- | --- | --- |
| 16. Medical teams fail to recognise the palliative needs of the child. | 5 | 4 | 3 | 2 | 1 |
| 17. The services available in the community are dependent upon where the child lives. | 5 | 4 | 3 | 2 | 1 |
| 18. Palliative care is only considered late in the child’s condition or in crisis management. | 5 | 4 | 3 | 2 | 1 |
| 19. Out patient appointments are prioritised in favour of these children and their families. | 5 | 4 | 3 | 2 | 1 |
| 20. Children and families suffer because of long delays in obtaining necessary equipment. | 5 | 4 | 3 | 2 | 1 |
| 21. Health services are under-resourced with respect to services required for these children and their families. | 5 | 4 | 3 | 2 | 1 |
| 22. Health professionals acknowledge parents’ expertise in the care of their child. | 5 | 4 | 3 | 2 | 1 |
| 23. Acute services are not aware of the range of services that are available to these children and their families in the community. | 5 | 4 | 3 | 2 | 1 |
| 24. Access to specialist palliative care services is readily available to these children if it is required. | 5 | 4 | 3 | 2 | 1 |
| 25. The lack of staff within services results in children not receiving the care they need. | 5 | 4 | 3 | 2 | 1 |
| 26. It is easy for parents to access the services their child needs. | 5 | 4 | 3 | 2 | 1 |
| 27. Families receive conflicting information about their child from different services. | 5 | 4 | 3 | 2 | 1 |
| 28. It is difficult to get a holistic view of the child’s needs because of the number of different health professionals and services involved in the child’s care. | 5 | 4 | 3 | 2 | 1 |
| 29. It is easy for parents to get information about the services that are available to their child. | 5 | 4 | 3 | 2 | 1 |
| 30. Insufficient funding results in the need to ration services to these children and their families. | 5 | 4 | 3 | 2 | 1 |
| 31. There is collaboration between the different services regarding the goals of care for these children. |  |  |  |  |  |
| 32. Parents are considered equal partners in the setting and prioritising of goals for their child’s care. |  |  |  |  |  |

Section 3

*This section relates to suggestions for improving the situation for children with life-limiting*

*neurodevelopmental disabilities and their families.*

Based upon the data collected from parents and the responses in round one of the survey the following issues were identified as the most important factors for improving the situation for this population of children and their families.

You are asked to do two things with this list of factors –

1. Please read and review all of the 18 factors on the list. Rank the factors in order of their potential to improve the current situation for children and their families. Assign a weight of one (1) to the factor you believe would make the most important contribution, two (2) to the second most important factor etc. until you have assigned a weight to all 18 factors.
2. Comment, in one or two statements, on any factor(s) that you wish. You may argue in favour of a factor, against a factor, or request clarification. Once again, brevity and clarity will facilitate analysis.

| **Weight** | **Improvement** | **Comment** |
| --- | --- | --- |
|  | - A greater level of communication between the different health professionals involved in the care of the child. - A key worker available to every family. - A single care plan that is used across all services. - A greater level of coordination and integration of the services involved in the care of the child. - A single point of contact for information for families. - Less bureaucracy surrounding family’s entitlements. - Access to specialist palliative care in a timely and efficient manner. - Parent held medical records. - A national directory of the services available to children and their families. - Improved education for community based health professionals. |  |

CONTINUED

| **Weight** | **Improvement** | **Comment** |
| --- | --- | --- |
|  | - A specialist paediatric palliative care consultant to act as a resource when required. - A formal coordinator of services for children with life-limiting disabilities in every HSE area. - Affording “medical priority” status to these children in Emergency and Outpatient departments. - Development of community based paediatric palliative care teams. - Improved respite facilities. - Less protracted ordering system for essential equipment. - Improved liaison between acute services and other service providers. - National standards of service and care. |  |

**Thank you for completing the second round of this Delphi survey.**

Please use the final sheet of the questionnaire if you would like to make any additional comments on the factors listed in section 3 of the questionnaire.

| Please use this space if you would like to provide any additional comments on the factors listed in Section 3. |
| --- |
